# Non-Invasive Transcutaneous Spinal DC Stimulation as a Neurorehabilitation ALS Therapy in Awake G93A Mice: The First Step to Clinical Translation

**DOI:** 10.3390/bioengineering9090441

**Published:** 2022-09-05

**Authors:** Morgan M. Highlander, Sherif M. Elbasiouny

**Affiliations:** 1 Department of Biomedical, Industrial, and Human Factors Engineering, College of Engineering and Computer Science, Wright State University, Dayton, OH 45435, USA; 2 Department of Neuroscience, Cell Biology, and Physiology, Boonshoft School of Medicine, College of Science and Mathematics, Wright State University, Dayton, OH 45435, USA

**Keywords:** tsDCS, spinal stimulation, DC stimulation, ALS, motoneuron, excitability, SOD1 G93A, electroceutical

## Abstract

Spinal direct current stimulation (sDCS) modulates motoneuron (MN) excitability beyond the stimulation period, making it a potential neurorehabilitation therapy for amyotrophic lateral sclerosis (ALS), a MN degenerative disease in which MN excitability dysfunction plays a critical and complex role. Recent evidence confirms induced changes in MN excitability via measured MN electrophysiological properties in the SOD1 ALS mouse during and following invasive subcutaneous sDCS (ssDCS). The first aim of our pilot study was to determine the clinical potential of these excitability changes at symptom onset (P90-P105) in ALS via a novel non-invasive *trans*cutaneous sDCS (tsDCS) treatment paradigm on un-anesthetized SOD1-G93A mice. The primary outcomes were motor function and survival. Unfortunately, skin damage avoidance limited the strength of applied stimulation intensity, likewise limiting measurable primary effects. The second aim of this study was to determine which orientation of stimulation (anodal vs cathodal, which are expected to have opposing effects) is beneficial vs harmful in ALS. Despite the lack of measured primary effects, strong trends in survival of the anodal stimulation group, combined with an analysis of survival variance and correlations among symptoms, suggest anodal stimulation is harmful at symptom onset. Therefore, cathodal stimulation may be beneficial at symptom onset if a higher stimulation intensity can be safely achieved via subcutaneously implanted electrodes or alternative methods. Importantly, the many logistical, physical, and stimulation parameters explored in developing this novel non-invasive treatment paradigm on unanesthetized mice provide insight into an appropriate and feasible methodology for future tsDCS study designs and potential clinical translation.

## 1. Introduction

Amyotrophic lateral sclerosis (ALS) is an adult-onset disease affecting both upper and lower motoneurons, with an incidence of 1 in 50,000 people [1]. Patient life expectancy is only 2–4 years from diagnosis [2]. Symptoms progress from localized reduced function or weakness (usually in a limb) to widespread motor function loss, then debilitating paralysis that ultimately leads to death. Current treatment options are very limited, with many side effects. Examining adjunctive treatments with the potential to improve the duration and quality of life for ALS patients is, therefore, a high priority.

The mechanisms of motoneuron (MN) degeneration in ALS are largely unknown, but MN excitability dysfunction is extensively documented throughout disease progression and tightly linked to disease pathogenesis. For instance, abnormal cell excitability is seen in all early MN disease changes [3,4,5]. Furthermore, abnormal excitability underlies spontaneous MN firings, which lead to muscle fasciculations occurring prior to paralysis and correlating with shortened patient survival in ALS [6]. Additionally, the only two drugs that are FDA-approved to treat ALS are Riluzole and Radicava, which moderately prolong survival [7,8,9] and are known to alter MN excitability. The former works by suppressing MN excitability, in part by reducing Na persistent inward currents (PICs) [10]; the latter by suppressing neuronal excitability indirectly [11]. Together, MN excitability dysfunction appears to play an unclear yet critical role throughout ALS. Furthermore, the effect of Riluzole suggests an important role for PICs in ALS.

Electroceuticals may have untapped potential to regulate neuronal excitability in ALS. These neurorehabilitation therapies use electrical fields to modulate the neuronal membrane potential and, thereby, the activation level of ion channels and overall cell excitability. Unlike pharmaceuticals, electroceuticals are physics-controlled and spatially targeted, which presents a different set of challenges. They have been shown in various clinical applications to effectively modify the neural activity for extended periods of time. For example, deep brain stimulation and transcranial DC stimulation are treatment options for Parkinson’s disease [12] and stroke [13], respectively. Likewise, spinal DC stimulation (sDCS) has been shown to modulate spinal neural circuitry during rehabilitation after spinal cord injury [14]. There are several cellular mechanisms by which sDCS specifically has been shown to modulate MN excitability. For example, sDCS was suggested by computational models to effectively regulate the dendritic PICs of MNs [15]. Additionally, recent in vivo studies show subcutaneous spinal DC stimulation (ssDCS) modulates measured electrophysiological properties, resulting in altered excitability of spinal MNs, during and beyond the stimulation period [16,17,18,19,20,21].

Despite the promising ability of sDCS to modulate MN excitability long-term, the complex and dynamic nature of the excitability dysregulation seen in ALS is only partially mapped, and additional factors are still under investigation. This makes the design of such a stimulation treatment paradigm equally complex and dynamic. Furthermore, the parameters of a potential electroceutical treatment (types, intensities, duration, etc.) will likely require a notable effort to determine and optimize. Significantly, the electrode–skin interface limits the maximal stimulation intensity for a non-invasive transcutaneous paradigm. Thus, the overarching goal of this pilot study was to look for preliminary evidence to assess the clinical potential of electroceutical therapy as an alternative treatment for ALS. Specifically, we used daily sessions of non-invasive transcutaneous spinal DC stimulation (tsDCS) with the goal of inducing lasting excitability changes in lumbar spinal MNs to affect motor function loss and survival in ALS via anodal (dorsal-to-ventral) or cathodal (ventral-to-dorsal) stimulation. Due to the physics of the polarizations induced across MNs in the stimulated region [15], it is expected that the cathodal and anodal orientations will have opposing effects (hyper or hypo-excitation), a phenomenon supported by measured MN changes in previous studies [16,19,20]. We hypothesized, therefore, that these opposing effects would make one orientation beneficial and the other harmful to symptom progression.

The tsDCS was applied to start at the clinically relevant symptom onset (when patients are diagnosed and begin treatment) in G93A mice, a standard transgenic animal model of ALS. Specifically, daily stimulation sessions lasting 30 min began at P90 and continued until P105 in three separate treatment groups: the anodal group, the cathodal group, and the sham control group. We examined the effect of the stimulation on motor function and survival (primary outcomes), as well as on the correlations between disease symptoms and survival (secondary outcomes). The latter allowed us to examine treatment effects on disease progression even in the absence of notable primary outcomes. Stimulation intensities are limited in non-invasive transcutaneous stimulation to avoid skin burns, which results in modest primary outcome treatment effects. However, the trends of primary outcomes combined with the correlation analysis suggest that anodal stimulation (which increases MN excitability [16,17]) tends to shorten G93A survival. Given that sDCS has demonstrated long-lasting modulation of spinal MN excitability in both computer models and in vivo electrophysiology studies, the current study is an important initial step toward exploring the clinical applicability of tsDCS. This study also provides valuable information on the technical considerations and challenges involved in non-invasive electrical stimulation interventions, thereby informing the development of clinically translatable (i.e., non- or minimally-invasive) paradigms for ALS treatment.

## 2. Materials and Methods

Three experimental groups of SOD1-G93A male transgenic mice were used to test the efficacy of non-invasive tsDCS as an alternative therapy for ALS: (1) anodal, (2) cathodal, and (3) sham. The anodal group received tsDCS with electrical field induced along the dorsal-to-ventral direction. The cathodal group received tsDCS with electrical field induced along the ventral-to-dorsal direction. The sham group was the control group, which had electrodes placed without electrical stimulation. Each group contained *n* = 13 SOD1-G93A transgenic male mice.

**Figure 1:**
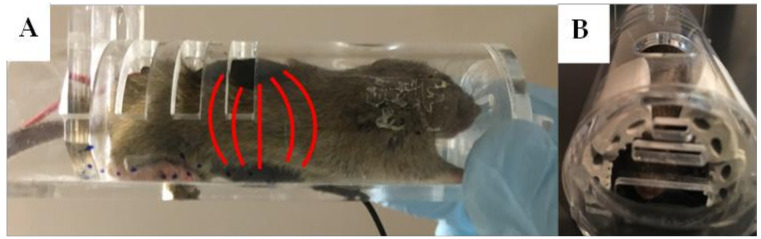
(**A**) Mounted saline-soaked sponge electrodes on mouse in plexiglass restrainer. tsDCS -induced electric field lines are illustrated by the red lines between the electrodes. (**B**) Foam added to restrainer to prevent access to electrode wires and calm mice.

For all groups, electrodes were placed over the lumbar region of the spinal cord, with one electrode on the back and an identical electrode on the abdomen (see Figure 1A). Electrodes were held firmly in place between the mouse and a plexiglass mouse restrainer. To prevent transient painful sensations when electrical current is switched on/off, a short (approximately 5 s) current ramp was used to turn the stimulation up to the desired intensity and then down to turn off the stimulation at the end of the session in the anodal and cathodal stimulation groups. Electrical stimulation sessions were 30 min in duration and were performed once daily, starting at symptom onset from P90 and continuing to P105. The sham group was restrained with mounted electrodes for the same duration but did not receive any stimulation.

All experiments and animal handling were performed in accordance to federal guidelines and with approval by the Institutional Animal Care and Use Committee (IACUC) at Wright State University (protocol #: AUP 1010).

Data collection was performed for three measures: weight, motor function, and survival. Weight is one predictor of ALS progression [22] because ALS animals normally lose weight over time as they are weakened by the disease and can no longer walk in their cage to reach food. Weight was recorded before symptom onset at P72, P73, P74, P75, P80, and P85. These ages were chosen for data collection because they are the ages at which the animals are acclimated to the motor function test, and they provide a good weight baseline for the adult mouse prior to symptom onset. Starting at P90, weight was recorded every other day until death of the animal.

**Figure 2:**
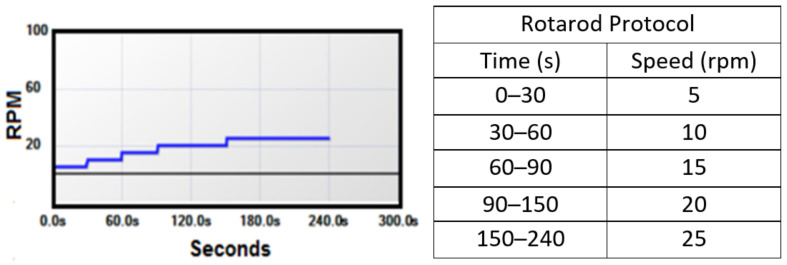
Rotarod motor function test protocol (rpm = rotations per minute).

Motor function was measured by a rotarod performance test. The animals were placed on the rotarod, where they walked to stay on the rod as it rotated at a series of increasing speeds (Figure 2). Acclimation to the rotarod occurred before symptom onset at P72, P73, P74, P75, and P80. By the end of the acclimation period, a non-transgenic or a pre-symptomatic mouse is able to complete the entire 240 s rotarod protocol and has a recorded motor function measure of or very near 1, meaning 100% of the protocol completed. As the disease progresses, performance of the transgenic mice gradually decreases from 1 until they are severely paralyzed and fall off the rod immediately. To ensure true motor function ability is measured, each mouse completes 3 trials on a given day, and the best trial is recorded as the animal’s motor function measure for that day. The rotarod test is performed at P85 and P90 for a baseline level (mostly = 1) and then every other day until the endpoint criteria are met. The endpoint of motor function data collection occurs when the mice score less than 0.05 on the rotarod—when they are falling as soon as the rod rotates far enough for gravity to pull them off.

For ethical reasons, survival cannot be recorded as the day when the mice die from the disease. Instead, our survival age is the day when the mice can no longer reach their food/water. This day is determined by a righting test, in which the mouse is placed on its back and given 30 s to turn over. If paralysis is so severe that the mouse cannot right itself within 30 s, it is humanely euthanized according to IACUC protocol, and its age is recorded for survival.

## 3. Results

### 3.1. Outcomes of Parameter and Methodology Exploration to Inform Future Studies

To our knowledge, tsDCS has not previously been performed on unanesthetized mice. Accordingly, several challenges arose in developing these novel experimental methods. This section discusses the results of the technical considerations faced in designing and executing this treatment paradigm in order to inform future studies to further explore the clinical potential of this electroceutical therapy in ALS or other applications.

### 3.2. Method of Restraint

To ensure secure electrode placement, maintain good contact with the skin, and prevent animals from chewing or scratching at the surface electrodes or connecting wires, the animals had to be restrained during stimulation. We used head-first plexiglass mouse restrainers (Figure 1). Most mice could turn themselves around in the restrainer, giving them access to the electrode wires and making it difficult to secure the electrodes in place. Thus, we attached small pieces of adhesive rubber foam to the interior of the restrainer on either side of the head area, as shown in Figure 1B. This calmed the animals by darkening their visual surroundings and prevented them from turning in the restrainers, thereby maintaining secure electrode placement and contact during tsDCS stimulation.

### 3.3. Electrode Design

**Figure 3:**
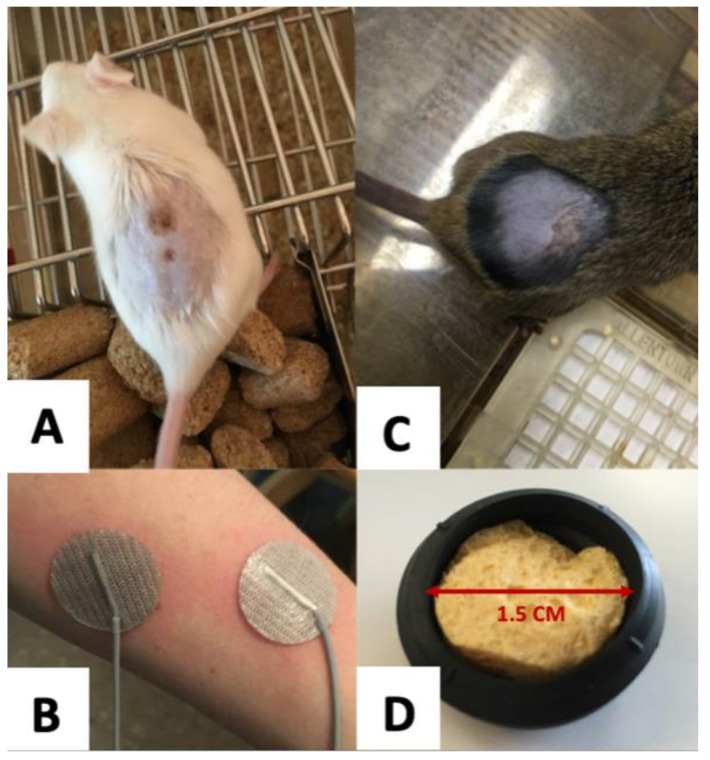
Electrodes and corresponding tissue damage. (**A**) Tissue damage from Ag-AgCl adhesive electrodes. (**B**) Ag-AgCl adhesive electrodes. (**C**) Tissue damage from saline-soaked sponge electrodes. (**D**) Homemade saline-soaked sponge electrode (also see Figure 1A for visual of the electrode size relative to the mouse).

Two types of electrodes commonly used with tsDCS are Ag-AgCl adhesive electrodes and saline-soaked sponge electrodes. We first tried Ag-AgCl adhesive electrodes (Figure 3B) in hopes that the adhesive quality would make them more suitable for awake mice. However, the adhesion was sometimes too weak, causing the thin electrodes to lose contact during stimulation. This resulted in chemical tissue damage from “hot spots” of current concentrated in small areas where contact remained (Figure 3A). At other times, the adhesion was too strong, significantly irritating the skin upon removal of the electrodes. Accordingly, we switched to saline-soaked sponge electrodes (Figure 3D), which provide a buffer between the charge-distributing metal and the skin of the animal. This successfully reduced the probability of chemical tissue damage (Figure 3C). Because we were unable to find commercially available sponge electrodes of appropriate size, we made the saline-soaked sponge electrodes at a specific diameter (1.5 cm) and thickness (approximately 0.75 cm), so they could be securely wedged between the animal and the plexiglass restrainers. The electrodes were made using Ag-AgCl wire mesh cut into a circle, covered by a thin sponge, and secured in a circular rubber housing. The sponge was saturated with saline before each stimulation session, and the electrodes were replaced every 2–3 sessions when they began to show visible signs of corrosion.

### 3.4. Skin Preparation

In order to achieve the necessary contact between electrode and skin, the mice were shaved. In order to avoid the induced stress and physiological changes from even a single dose of anesthesia, we shaved the awake animals on their back by holding their tail and encouraging them to grip the cage, so they could be fully extended for a close shave. The abdomens were shaved while the animals were in the scruff. Mice did not regrow hair for the duration of the experiment, so a single shaving was sufficient. However, all mice in the study had to be separated from their littermates and housed individually to prevent irritation of the shaved area from fighting or over-grooming between littermates.

### 3.5. Current Density and Total Charge Delivered

For prevention of tissue damage, current density and total charge delivered in a session must be maintained within safe limits. The most common current density value for tDCS performed in studies on humans is 0.29 A/m^2^ [23], with a corresponding total charge delivered ranging from 0.0087 to 0.031 C/cm^2^ (see Table 1). A comparable study of sDCS in anesthetized rats, using a current density of 12.7 A/m^2^ for 15 min sessions, reported a maximal total charge of 1.15 C/cm^2^ [24]. However, this study used implanted electrodes (that is, ssDCS), allowing the total charge delivered to be higher without risk of tissue damage from electrode–skin contact. The maximal reported current density was from a study that performed tsDCS on anesthetized mice at current densities up to 38.2 A/m^2^ for a duration of 3 min [25]. The corresponding total charge delivered was 0.6876 C/cm^2^.

Based on the above information, we began initial testing of our tsDCS stimulation at a current density of 11.32 A/m^2^ in a small group of animals. For our duration of 30 min, our total charge delivered was 2.0 C/cm^2^, nearly double the reported maximum of 1.15 C/cm^2^. When delivered by our adhesive Ag-AgCl electrodes, this intensity caused significant tissue damage in the form of small skin burns under the electrodes. The frequency and severity of the tissue damage were reduced with the use of our homemade saline-soaked sponge electrodes, but nonetheless, the stimulation intensity had to be lowered until tissue damage was completely eliminated. Once we confirmed safe parameters for burn-free stimulation sessions on our testing animals, we then recruited the experimental animals, which were randomly assigned to the three groups (anodal, cathodal, and sham) for the 16 daily treatments. Therefore, all data reported are from the experimental animals with lowered, burn-free stimulation intensity. The maximal safe current density value we were able to use was 2.83 A/m^2^, corresponding to a current-controlled stimulation of 0.5 mA with our 1.5 cm diameter round electrodes and a total charge, delivered over 30 min, of 0.51 C/cm^2^. This total charge delivered is similar to the 0.6876 C/cm^2^ used in Ahmed’s comparable 2011 transcutaneous study [25] and is, unsurprisingly, much smaller than the 1.15 C/cm^2^ used in Aguilar’s comparable 2011 study with implanted electrodes [24].

The selection of 30 min for our session duration was based on previous literature showing long-term effects following 3–20 min of tDCS (see Table 1). Although the duration of tsDCS effects increases as stimulation duration increases, we limited our stimulation to 30 min to avoid increased stress on mice from prolonged restraint and the increased likelihood of tissue damage, as the total charge delivered also increases with stimulation duration. Therefore, 30 min was a reasonable balance among these considerations.

### 3.6. Effects on Disease Progression: Primary and Secondary Outcomes

#### 3.6.1. Weight

**Figure 4:**
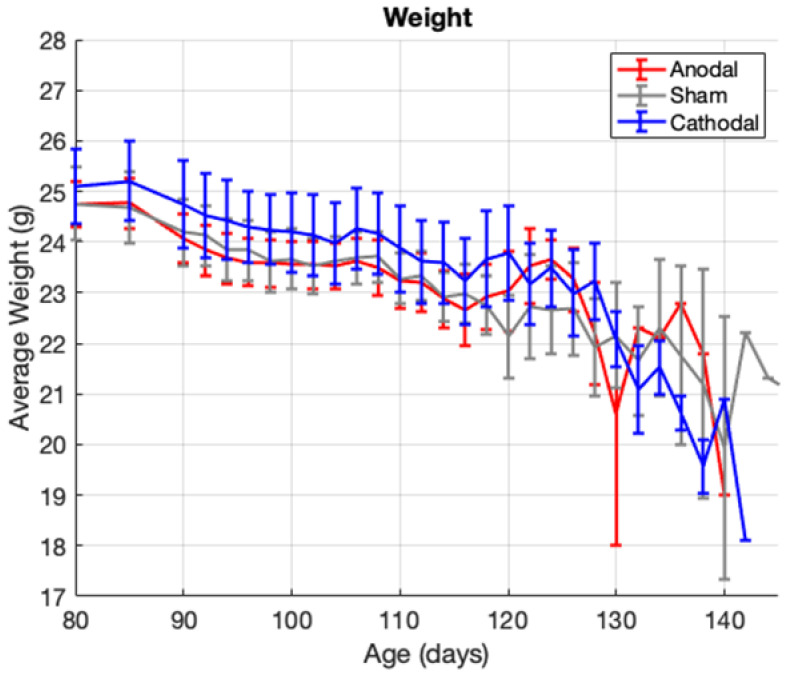
Group average weight vs. age shown with standard error bars.

The weight of mice in the cathodal group was significantly greater overall than the weight of mice in the sham group (Dunnet’s post hoc comparison, *p* = 0.0261). However, this difference in weight cannot be attributed to the treatment because the cathodal group trended towards a higher baseline weight before the treatment. Additionally, there were no significant weight differences between groups at any given age (two-way ANOVA, all *p*’s >> 0.05), and the overall rate of weight loss was not different between the groups (one-way ANCOVA, *p* = 0.6442). These weight data are presented in Figure 4 and show a lack of anodal or cathodal treatment effect on weight loss. It should also be noted that the standard error of the weight data is very large, illustrating the high variability of baseline weight and weight changes post-symptom-onset in this mouse model. This indicates the need for careful consideration of the potential confounding effects of weight on the other symptoms of motor function and survival.

#### 3.6.2. Motor Function

**Figure 5:**
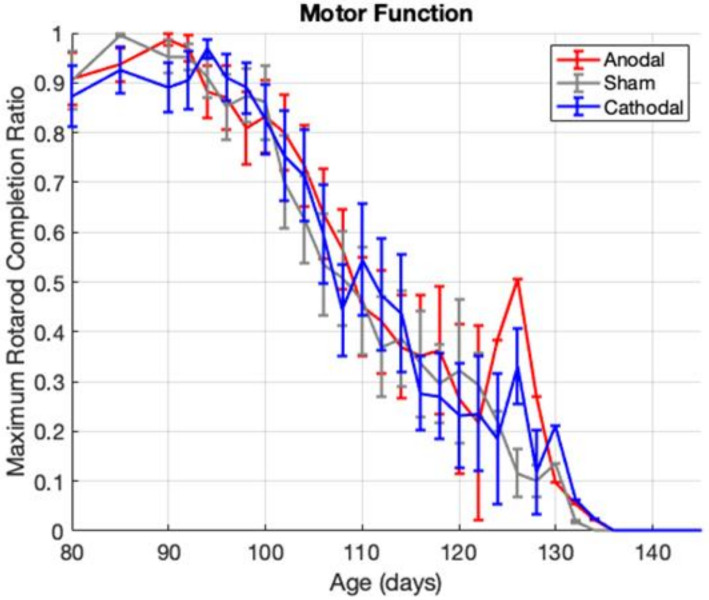
Group average motor function score vs age shown with standard error bars. The averages show sharp and dramatic changes in the later ages as more animals meet the end-stage running criteria and thus fewer animals remain in each group to contribute to the group average.

Motor function scores were not different between the anodal, cathodal, and sham treatment groups overall (one-way ANOVA, *p* = 0.4113) or at any given age (two-way ANOVA, all *p*’s >> 0.05). The rate of motor function decline was also not different between treatment groups (one-way ANCOVA, *p* = 0.9589). The motor function data are presented in Figure 5 and show a lack of anodal or cathodal treatment effect on motor function.

**Figure 6:**
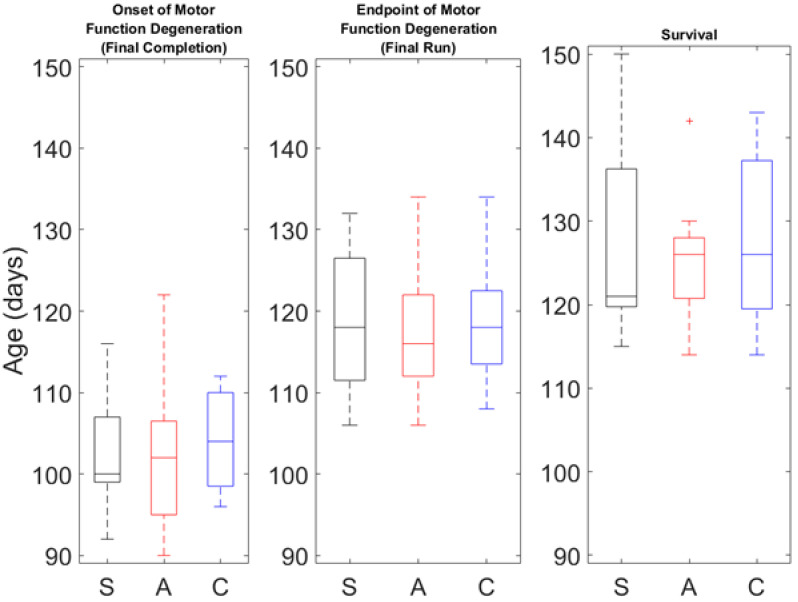
Box plots of survival and motor function time point comparisons where S = sham, A = anodal, and C = cathodal. Note the single outlier shown in the survival of the anodal group. Furthermore, note the much smaller inner quartile ranges of the anodal group as compared to the sham and cathodal groups.

It is also interesting to consider possible treatment effects on the onset and endpoint ages of motor function decline. The onset age of motor function decline is considered the day on which the animal first fails to complete the entire rotarod protocol, called the first failure. Endpoint age of motor function decline is considered the day on which the animal meets the rotarod end-stage criteria (cannot stay on the rod for more than 5% of the protocol), called the final failure. There were no differences between treatment groups for the first failure or final failure, as shown in Figure 6. Mean and standard deviation values are presented in Table 2.

#### 3.6.3. Survival

**Figure 7:**
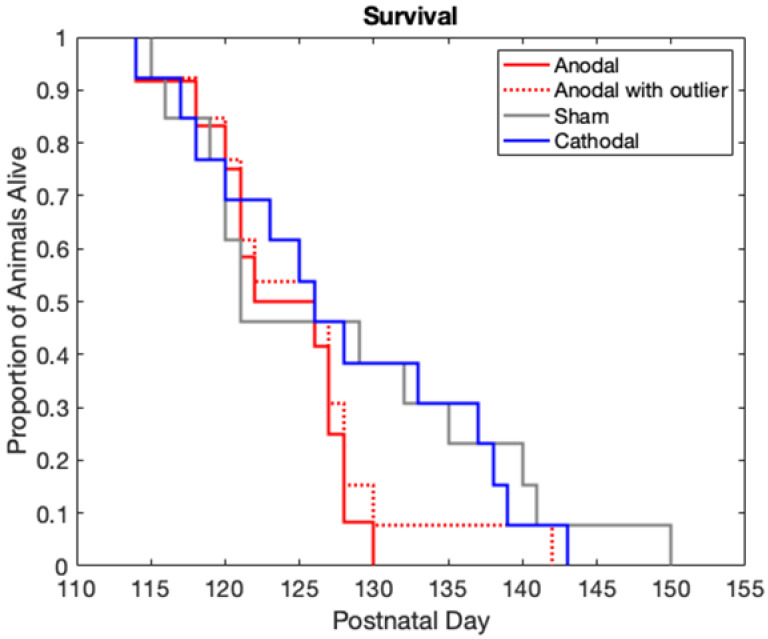
Survival vs. age by group. Note the high number of animals who die around P125–P130 in the solid red anodal treatment group.

The Kaplan–Meier graph in Figure 7 shows G93A survival by the proportion of animals alive in each group at a given age. The anodal group had a strong trend towards a negative effect on survival, illustrated by the death of more animals in the anodal group around P125–P140. There was a single outlier (see Figure 6) in the anodal group that survived much longer than the other animals in the group. For completeness, the survival data with this outlier included are shown by the dashed line in Figure 7, while the solid line represents the survival data with the outlier removed. While there were no statistical differences in survival between the groups with or without the outlier, its removal resulted in a strong trend towards reduced survival in the anodal group. Mean survival data are presented graphically in Figure 6 and numerically in Table 2.

#### 3.6.4. Correlations

The lack of notable treatment effects on motor function and survival could be the result of suboptimal parameter values, such as the limited current intensity to avoid skin burns, and therefore does not strongly indicate a general lack of treatment potential for sDCS in ALS. Thus, we looked for more subtle treatment effects in the correlations of symptoms to form hypotheses for future studies exploring the clinical potential of tsDCS. This analysis provided us with additional insights on how tsDCS treatment affected known relationships among symptoms.

**Figure 8:**
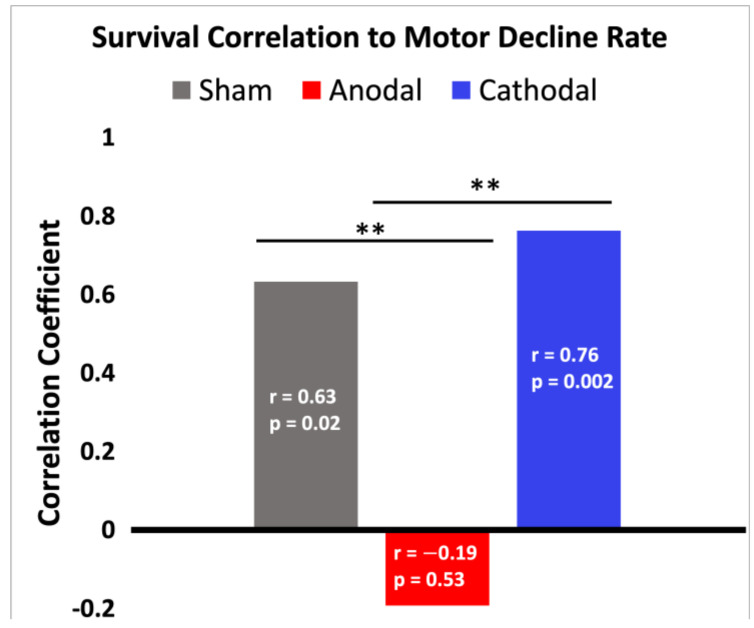
Correlation coefficient comparisons. In the bars, the correlation coefficient r is displayed with its corresponding *p*-value as listed in Table 3. *p* < 0.05 rejects the null hypothesis that r = 0 and indicates a significant correlation. To compare the coefficients between groups, two-tailed fisher’s r-to-z transformation was used. ** indicates the correlation coefficients are significantly different at an alpha of 0.05.

Specifically, we focused on the relationship between the rate of motor function decline and survival during ALS progression in G93A mice (see Figure 8). Normally, a G93A SOD1 mouse with a faster rate of motor function decline would tend to have shorter survival and vice versa. This expected correlation is strong and positive in the sham (r = 0.6328, *p* = 0.0203) and cathodal (r = 0.7631, *p* = 0.0024) treatment groups, indicating that cathodal stimulation did not disrupt this relationship. Interestingly, anodal stimulation did disrupt this relationship and showed a negative correlation between motor function and survival (r = −0.1923), although it was not statistically different from zero (*p* = 0.5290). This lack of correlation between motor function decline and survival in the anodal treatment group suggests a deviation from normal symptom progression due to the applied stimulation. Additionally, as shown in Figure 6 by the anodal group’s much more compact box and whisker plot, there is a lower variance in survival in the anodal treatment group as compared to the sham (two sample F-test for equal variance, *p* = 0.0051). The smaller survival variance and trend toward a lower mean survival in the anodal group, combined with the group’s loss of correlation between survival and rate of motor decline, together suggest a negative anodal stimulation effect on survival. We hypothesize that this effect will be much larger and likely result in a statistically significant decrease in mean survival if a stronger stimulation intensity can be safely achieved.

As baseline weight and the rate of weight loss during the symptomatic period could influence both motor function performance and survival, we examined the relationships between baseline weight and rate of weight loss with survival and motor function decline. Baseline weight was taken as the average of recorded weights before symptom onset (P72, 73, 74, 75, 80, and 85). We found no correlation between baseline weight and survival within any treatment group (all *p*’s > 0.3, see Table 3) and therefore concluded that baseline weight does not predict survival (or vice versa). Similarly, we found no correlation between baseline weight and rate of motor function decline in any treatment group (all *p*’s > 0.1, see Table 3) and concluded that baseline weight does not predict the rate of motor function decline (or vice versa). These conclusions are important because the cathodal treatment group did have a higher baseline weight than the other groups, but we have shown no biasing effects of a higher baseline weight on motor function of survival.

Additionally, and as expected, the correlation between weight loss rate (taken from P106-death) and survival trended positive in all groups (r = 0.3913, 0.3912, and 0.5326 for sham, anodal, and cathodal, respectively). This suggests that animals that die sooner tend to have a more rapid weight loss and vice versa. However, these correlation coefficients could not be statistically differentiated from zero for any group (all *p*’s > 0.06, see Table 3), likely because of weight’s high variability. Importantly, the correlation coefficients were not statistically different between any two groups (two-tailed fisher’s r-to-z transformation to compare correlation coefficients, all *p*’s > 0.65), indicating no detectable treatment effects on the correlations of weight loss rate and survival at these stimulation intensities.

Finally, and as expected, the correlation between weight loss rate (taken from P106-death) and motor function decline rate trend positive in the sham and cathodal groups (r = 0.5005 and 0.3408 for sham and cathodal, respectively). This suggests that animals that have a more rapid weight loss rate also tend to have a more rapid motor function decline. However, this correlation unexpectedly trends negatively in the anodal group (r = −0.4040). None of these correlation coefficients can statistically be differentiated from zero (all *p*’s > 0.08, see Table 3), likely because of weight’s high variability. However, it is interesting to note that the anodal correlation coefficient is statistically different at alpha = 0.05 from the sham group and at alpha = 0.1 from the cathodal group (two-tailed fisher’s r-to-z transformation to compare correlation coefficients, *p* = 0.0285 and *p* = 0.0801, respectively). With no statistical difference (or strong trend towards a difference) between groups in the motor function decline or weight data, we can draw no specific conclusions about the mechanism of this unexpected effect of anodal stimulation. However, this change from the expected symptom correlation does further support the notion that the anodal stimulation is disrupting symptom progression in a complex manner and should be studied at larger stimulation intensities.

## 4. Discussion

The purposes of this pilot study were to (1) make an initial assessment of both the treatment potential and the technical challenges of non-invasive electroceutical neurorehabilitation therapy in ALS and (2) explore the harmful vs. beneficial orientations of the stimulation polarity. We chose the clinically relevant symptom-onset stage in the G93A mouse model of ALS, as this corresponds to the start of therapy in patients. Our study describes the technical challenges and considerations needed for the clinical applicability of totally non-invasive tsDCS stimulation. Although avoidance of skin burns limited the intensity of delivered tsDCS current, the disruption of known correlations among disease symptoms confirmed that our stimulation intensities did subtly alter normal disease progression, even in the absence of measurable primary outcomes. Analysis of survival variance and correlations among symptoms suggest that anodal stimulation has detrimental effects on the survival of G93A mice, a hypothesis that would remain to be confirmed at higher tsDCS intensities. Anodal stimulation increases MN excitability [16,17]. Furthermore, classical excitotoxicity theory predicts that hyperexcitability is a disease mechanism that kills MNs [7], and MN hyperexcitability has been reported in symptomatic SOD mice [35]. Taken together, the conclusion drawn from our data that anodal stimulation has detrimental effects on SOD mice survival has supporting evidence in the literature and is highly plausible. As such, this study has generated a testable hypothesis on the effect of tsDCS stimulation on the survival of G93A mice and is expected to greatly inform the design of future non-invasive tsDCS and likely other stimulation paradigms such as ssDCS.

### 4.1. Physics of tsDCS

The uniform electrical field generated by tsDCS affects motoneuron excitability in a complex manner. Conceptually, the polarization effect has been documented [36] and modeled [15]. The electrical field, by definition, is the voltage gradient created when current is passed between a stimulating electrode and a reference electrode. The induced voltage gradient will cause ionic current to enter the cell through channels closest to the stimulating electrode and exit the cell through channels closest to the reference electrode. The net effect of this current flow is an individual polarization gradient felt across all structures in the field.

**Figure 9:**
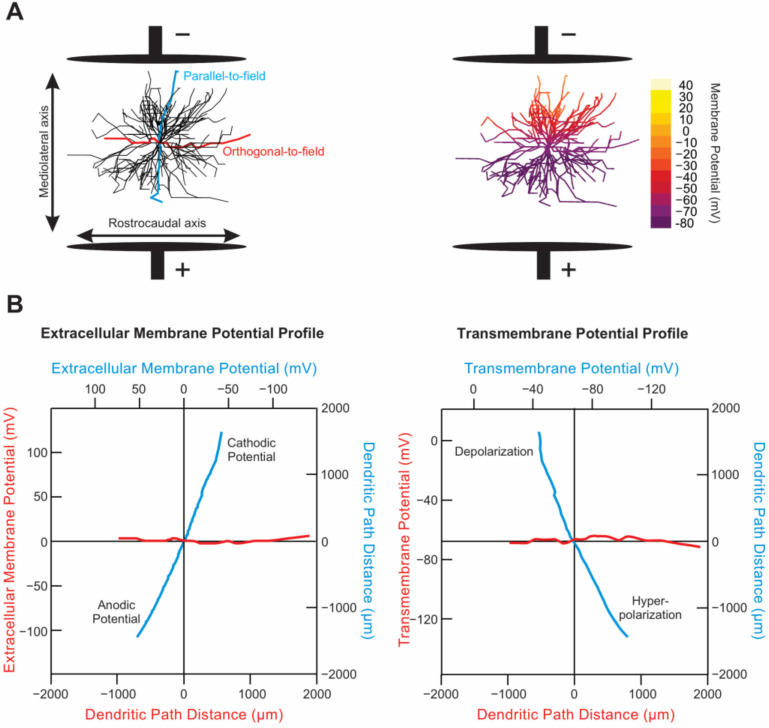
The spatial distribution of membrane polarization along the dendritic membrane for a MN placed in a DC electric field. (**A**) electrical field applied along the mediolateral axis of the SC and generated by current flow between 2 plate electrodes was simulated (left). Dendritic segments aligned orthogonal (red) and parallel (blue) to the field axis are shown. Spatial distribution of the dendritic transmembrane potential caused by the imposed field is shown at right. (**B**) extracellular membrane potential (left) and transmembrane potential (right) profiles formed by the imposed electrical field along the dendritic segments shown in A at steady state and during no activation of synaptic input. The spatial arrangement of the dendritic segments is preserved relative to the imposed field. x-Axis and y-axis labels have the same color as the trace. Soma location is at the origin. (adapted with permission from [15]).

Each motoneuron experiences local membrane hyperpolarization on parts of the cell oriented towards the stimulating electrode. The stimulating electrode is called the anode and is labeled as the positive (+) electrode. Hyperpolarization is greatest in cell compartments oriented closest to the anode and decreases linearly towards the center of the cell geometry, where the polarization effect is zero or neutral. Beyond the center of the geometry, the motoneuron experiences depolarization that increases linearly towards the (−) cathode. This phenomenon is illustrated by the modeled motoneuron in Figure 9 [15]. Geometry extending perpendicular to the electrical field will experience nominal polarization (red lines in the bottom panels of Figure 9), and thus we only need to consider the polarization gradient in the direction of the induced field. Multiple studies have experimentally confirmed the polarization gradient generated across motoneurons in tsDCS [37,38,39,40,41,42].

Due to the strong (albeit imperfect) geometric symmetry of motoneurons, the soma of a motoneuron is mildly polarized, while its dendrites, especially distal ones, are strongly polarized by tsDCS. However, the net excitability of each motoneuron may still be significantly affected by the activation/suppression of PIC channels on its highly polarized dendrites or by the polarization of its motor axon, depending on its orientation relative to the field [39,42]. The network effect of tsDCS becomes even more complex when considering stimulation effects on synaptic activity, which are poorly understood.

Despite such complexity, many similar electrical stimulation techniques, such as deep brain stimulation (DBS) and functional electrical stimulation (FES), became clinically successful once their paradigms were optimized to achieve the desired effect, even though the exact neurophysiology of the techniques remains to be fully understood. Thus, although tsDCS is a relatively new technique with complex network effects, the current preliminary evidence suggests it has potential relevance in clinical applications where spinal motoneuron electrical activity is of interest.

Furthermore, the appropriateness of such an electroceutical therapy as an ALS treatment is enhanced by the known physics of the paradigm, which dictates a larger stimulation effect on larger objects, allowing us to leverage most of the treatment effect on the large motoneurons, which are also the most susceptible to degeneration in ALS. Furthermore, large, vulnerable MNs tend to become even larger in ALS [43], causing them to be polarized even further by the applied stimulation. This physics is well known and has also been modeled over the MN, as shown in Figure 9 [15]. Unfortunately, starting our stimulation at symptom onset means that most of the large MNs had already degenerated. Thus, targeting an earlier time period to stimulate the large MNs before they die could greatly increase the stimulation effect. This, in turn, could perhaps delay the onset of degeneration of the large MNs and ultimately have a much greater effect on disease progression. However, it will not be clinically relevant as long as a diagnosis in human patients takes place when symptoms emerge. Thus, until an earlier diagnosis of ALS is possible, it is important to optimize the treatment effects at symptom onset.

### 4.2. Anodal Stimulation May Be Harmful to Survival

Our results show that non-invasive anodal tsDCS starting at symptom onset had a strong trend towards shorter survival of G93A mice. This was further supported by the significant change in correlation between motor function decline and survival, as well as the lower variance in survival for the anodal group. Importantly, these results are in agreement with a large body of literature on the classical excitotoxicity theory, which predicts hyperexcitability as a disease mechanism that kills MNs (reviewed in [7]). As spinal anodal stimulation increases MN excitability [16,17] with effects that could be sustained for weeks [18], and MN hyperexcitability has been reported in symptomatic SOD mice [35], anodal stimulation could therefore accelerate the neurodegeneration process and lead to shortened survival of SOD mice. Interestingly, the negative anodal survival trend contradicts the prediction that anodal stimulation would have beneficial effects on G93A mice [20]. However, many factors could explain this discrepancy between this prediction by Baczyk, Krutki and Zytnicki [20] and our results: (1) **Timepoint**: In order to be clinically relevant, our study was completed at symptom onset (P90–P105), whereas Baczyk, Krutki and Zytnicki [20] study targeted a pre-symptomatic timepoint (around P50–P60). It is highly likely that excitability changes are dynamic throughout disease progression [44]. Therefore, increasing MN excitability with anodal stimulation could be beneficial earlier in the disease but harmful by symptom onset. Furthermore, the prediction of Baczyk, Krutki and Zytnicki [20] of beneficial anodal effects in ALS was not based on survival or behavioral experiments but rather was based on cellular data that anodal stimulation enhanced synaptic inputs to MNs and evoked MN hyperexcitability, along with the prediction that these effects would have a beneficial treatment outcome. Extrapolating the individual MN effects to predict the overall treatment outcome relies on filling gaps in the field’s knowledge of the import of the hypo- and hyperexcitability seen throughout disease progression—that is to say, which is the disease mechanism and which is the protective, compensatory mechanism. This theory is still highly debated and largely unknown. (2) **Anesthesia/PICs**: Data from the study on individual MNs measured by Baczyk, Krutki and Zytnicki [20] were collected in vivo with the animal deeply anesthetized and therefore with suppressed persistent inward currents (PICs). Known to be dysregulated in ALS [35,44] and a major component of MN excitability, the PICs are an important target for studying and/or modifying MN excitability. Due to the physics of sDCS-induced polarization across MNs (see Figure 9), PICs on the dendrites are likely greatly modulated by sDCS and thereby largely impact the net effect of the stimulation. Thus, anodal stimulation, which enhances MN excitability, most likely increases the dendritic PICs greatly. As our animals are not anesthetized, their PIC activity is preserved. This allows us to observe results of further enhancement of PICs by anodal sDCS, which could have detrimental effects on survival. In support of this scenario, the drug Riluzole (one of the only two FDA-approved treatments for ALS) extends survival, and is known to reduce PICs, which together support the possibility that enhancement of PICs would be detrimental [10].

One could argue that the adverse effects of anodal stimulation could be due to its effects on other internal organs due to the electrode’s size and placement on the abdomen and the back; however, the evidence does not support that. First, to avoid skin damage, the charge density and total charge of tsDCS stimulation we used in the present study were low. This low-intensity stimulation, applied non-invasively, becomes even more attenuated by the fat and skin. Thus, the stimulation intensity that might have reached internal organs is very low. Second, charge density and the total charge delivered, not electrode size, are the parameters that correlate with the strength of stimulation and its effects. In this study, both parameters were under safe limits (see Table 1). Third, the electrodes in the present study were placed very carefully to only target the lumbar region of the spinal cord, which minimizes current spread to other internal organs. Fourth, in other studies in which tsDCS stimulation was applied in an orientation similar to the present study (via electrodes placed on the abdomen and the back) and with comparable total charge [25], no organ stimulation effects were reported. Last, as mice have a high, fast metabolism, any illness reflects on the mouse’s weight very quickly. Thus, weight is a good indicator of overall mouse health that is often used in animal protocols to determine when animals should be excluded from a study due to illness. With no differences in weight seen in the treatment groups compared to the control group, this suggests that tsDCS stimulation did not negatively affect organ function of stimulated mice. Collectively, the adverse effects of anodal stimulation do not appear to result from the stimulation of other internal organs.

### 4.3. Weaker Cathodal Effect

It has been widely shown that anodal stimulation has a larger amplitude and longer lasting functional effects than cathodal stimulation [14,45,46]. Baczyk et al. have also shown stronger and more consistent anodal stimulation effects on MN electrophysiological properties than cathodal stimulation [16,17,18,19,20], and they have shown these effects in the G93A mouse model [20]. Thus, knowing that anodal stimulation has larger magnitude effects on MN excitability modulation, and considering the trend towards shorter anodal survival in our data, we hypothesize that cathodal stimulation has the potential to prolong survival when applied at symptom onset, but with the use of a stronger stimulation intensity than used in this study. This hypothesis on cathodal effects is supported in that anodal and cathodal stimulation involve reversed polarity and reversed current flow, and their effects on biological tissue are, therefore, usually reversed. The maximal intensity used in our study was greatly limited by the technical challenges inherent to performing this novel experimental paradigm on awake mice. We made great progress in finding stimulation parameters that could be safely performed in a true non-invasive, clinically relevant paradigm. However, we were limited in the total charge we could deliver by the highly resistant electrode–skin interface, which was further exacerbated by the movements of the awake mice. While a major goal of this study was to be entirely non-invasive, we have shown that this comes at the great sacrifice of charge density/current intensity. The need to limit the strength of polarization ultimately limited the ability to affect individual disease symptoms. Subcutaneously implanted electrodes could be a solution, but this would no longer be a fully non-invasive intervention. If subcutaneous stimulation is shown to have the potential to slow disease progression in the mouse model, two major outcomes would be achieved. (1) Similar studies could be conducted on humans, where the electrode–skin interface could prove more suitable to achieve a transcutaneous paradigm (it would be easier and more stable to secure electrodes to a cooperating human subject for a daily treatment period than to a mouse). (2) The beneficial vs. harmful effects of anodal vs. cathodal stimulation at specific disease stages, including at the symptom-onset stage, could be confirmed. If the conclusions from such studies were considered together with stimulation-induced excitability changes which have previously been measured and modeled at the cellular level in the same disease stages, we could gain additional significant, novel insights into the whole-network effect of increasing/decreasing motoneuron excitability. This information could be vital in sorting out the great hyper/hypo-excitability debate in the field of ALS.

### 4.4. Design Revisions: Secondary Study Outcomes and Clinical Relevance

Despite the mouse’s small size, tsDCS stimulation can be successfully delivered in normal mice [47] and in spastic mice after spinal cord injury [48], which inspired successful tsDCS stimulation testing in spastic human patients [49]. Because truly non-invasive spinal electrical stimulation had never been performed on awake ALS mice, we anticipated many technical challenges. The most limiting technical challenge was tissue damage from current hot spots at the electrode–skin interface, which forced us to lower the intensity of the stimulation. We believed our maximum current density was still strong enough to affect MN excitability because it was greater than many other studies that reported excitability changes. However, the lack of significant treatment effects in any of the symptoms suggests that our current density was too low to have a measurable harmful or beneficial effect on symptom progression. The high variability of symptoms in all groups (and in all cases of ALS, animal or human) may contribute to the lack of measurable effects and likely indicates the need for higher stimulation intensities than anticipated by non-ALS studies to adequately impact symptom progression.

In order to test our hypothesis that the anodal stimulation is negatively affecting disease progression, design revisions would be needed to increase the intensity or efficacy of stimulation. One way is to use grid electrodes that steer the electric current through the tissue more effectively than two-electrode configurations [50]. Another way would be to implant electrodes to avoid tissue damage and raise the charge density of stimulation. If wireless electrodes could be implanted, this could allow us to increase the duration of stimulation by avoiding restraint of the animals and thereby increasing the total charge delivered. Additionally, an increase in total charge delivered over the life of the animal could be achieved by extending the period of stimulation beyond 16 days. To maintain clinical relevance, we could still begin stimulation at symptom onset (P90) but continue the daily stimulation until death.

Our work highlights the potential skin damage that could result from non-invasive tsDCS stimulation if the stimulation intensity is high. In that regard, algorithms that evaluate the impact of electrical stimulation parameters on living cells would be very useful. Such evaluation of the effects of cortical electrical stimulation is available [51,52], and a similar evaluation for spinal cord stimulation would be of great assistance in the development of spinal cord electroceuticals.

### 4.5. Sex Effects

The present study was conducted on male mice only; thus, the effects of our tsDCS stimulation protocol on female G93A mice are unknown. Because sex is a biological variable in ALS (males are more commonly and aggressively affected by the disease than females [53]), and we wanted to control for sex effects on the results, male mice were only used in this pilot study. However, given the greater aggressiveness of the disease in males than females, we predict that tsDCS stimulation might have similar, or potentially larger, effects on female G93A mice.

## 5. Conclusions

Comparing the expected correlations of symptoms revealed an anodal treatment effect that indicates the stimulation likely did disrupt normal disease progression at a level not measurable by our individual symptoms. Specifically, the anodal treatment group’s rate of motor function decline was interestingly not correlated to survival. Furthermore, while not significant, the largest effect on an individual symptom was the trend towards a negative anodal effect on survival. The anodal group survival also had a lower variance than the sham control group, further suggesting a treatment effect. Combining this evidence, we predict that, at a higher intensity, the anodal stimulation at symptom onset would have a harmful effect on survival and overall disease progression.

The cathodal treatment group did not show any strong trends towards a treatment effect. We predict that, at a higher stimulation intensity, we could elicit a treatment effect opposite to the predicted anodal effect. It is consistent with the literature for the cathodal stimulation effect to be smaller than the corresponding anodal effect at the same intensity. This means we predict the effects to be opposing but not equal in magnitude. We predict a higher intensity of cathodal stimulation would have a beneficial effect on symptom progression, albeit smaller than the negative anodal effect. Implanted electrodes would be the most logical next step to test these hypotheses.

Importantly, we explored and optimized a large variety of methodologies for non-invasive stimulation on awake mice, including methods of restraint, skin preparation, electrode composition, positioning, and securing. Specifically, we sought to minimize stress and maximize safety for the animals. We also optimized the size, shape, and type of electrode for the given paradigm and maximized the physical symmetry of the electrode size and placement to reduce variability between the anodal and cathodal-induced electrical fields. Additionally, we explored a large electrical stimulation parameter space (current amplitude, stimulation duration, total charge delivered, surface area of electrodes, ramp-up and ramp-down rate to start and end stimulation) to narrow down the maximal safety thresholds for transcutaneous stimulation in mice. Optimizing these parameters and defining their suitable ranges is extremely important to inform future studies if the field is to make progress toward determining the full potential of electroceutical therapy as a clinically relevant neurorehabilitation treatment and its potential to be non- or minimally invasive (i.e., trans- or subcutaneous).

## Figures and Tables

**Table 1:** Long-lasting stimulation effects from previous literature [25,26,27,28,29,30,31,32,33,34].

Author	Technique	Density (A/m^2^)	Stimulation Duration (min)	Total Charge Delivered (C/cm^2^)	Effect Duration (min)
Nitsche 2000 [27]	tDCS	0.29	5	0.0087	5
Nitsche 2003 [26]	tDCS	0.29	9	0.01566	60
Lang 2004 [29]	tDCS	0.29	10	0.0174	40
Ardolino 2005 [30]	tDCS	0.43	10	0.0258	60
Nitsche 2005 [28]	tDCS	0.29	9–13	0.02262	40
Quartarone 2007 [31]	tDCS	0.29	7	0.01218	10
Monte 2010 [32]	tDCS	0.29	9	0.01566	60
Monte 2010 [32]	tDCS	0.29	18	0.03132	90
Aguilar 2011 [24]	sDCS (implanted)	12.7	15	1.15	N/A
Ahmed 2011 [25]	tsDCS	0.64–38.2	3	0.6876	20
Lazzaro 2012 [33]	tDCS	0.29	20	0.0348	180
Jankowska 2017 [34]	Epidural sDC		10		60

**Table 2:** Averages with standard deviation for first failure (onset of motor function degeneration), final failure (endpoint of motor function degeneration) and survival (age of failed righting test). The variance of the anodal survival is significantly smaller than the variance of the sham control group at an alpha of 0.05. Furthermore, note the smaller, albeit not significantly different, anodal survival mean.

Treatment Group	Final Completion		Final Run		Survival	
Sham	102.33 ± 6.60	(*n* = 12)	118.31 ± 9.09	(*n* = 13)	127.62 ± 11.11	(*n* = 13)
Anodal	102.31 ± 8.94	(*n* = 13)	117.38 ± 7.27	(*n* = 13)	123.50 ± 4.87	(*n* = 12)
Cathodal	104.18 ± 5.96	(*n* = 11)	118.77 ± 7.24	(*n* = 13)	127.77 ± 9.44	(*n* = 13)

**Table 3:** Correlation coefficients (r) for each treatment group and the corresponding *p* value indicating whether the correlation is significantly different from 0. Coefficients of particular interest and *p*-values significant at an alpha of 0.05 are shown in bold text. The interesting deviations of the anodal group are highlighted in light yellow.

Group	Correlation Coefficient (r)	*p*-Value *H*_*alt*_: r ≠ 0
Motor Decline and Survival		
S	**0.6328**	**0.0203**
A	**−0.1923**	0.5290
C	**0.7631**	**0.0024**
Baseline Weight and Survival		
S	−0.2035	0.5050
A	0.3094	0.3037
C	0.1448	0.6370
Baseline Weight and Motor Function Decline		
S	−0.1392	0.6501
A	−0.4629	0.1112
C	0.3559	0.2327
Weight Loss Rate and Survival		
S	0.3914	0.1860
A	0.3912	0.1862
C	0.5326	0.0609
Weight Loss Rate and Motor Function Decline		
S	**0.5005**	0.0815
A	**−0.4040**	0.1709
C	**0.3408**	0.2544

## Data Availability

The data presented in this study are available upon reasonable request from the corresponding author.
